# Husband and Wife Perspectives on Farm Household Decision-making Authority and Evidence on Intra-household Accord in Rural Tanzania

**DOI:** 10.1016/j.worlddev.2016.09.005

**Published:** 2017-02

**Authors:** C. Leigh Anderson, Travis W. Reynolds, Mary Kay Gugerty

**Affiliations:** a University of Washington, Seattle, USA; b Colby College, Waterville, USA

**Keywords:** decision-making, authority, farm households, women

## Abstract

We use OLS and logistic regression to investigate variation in husband and wife perspectives on the division of authority over agriculture-related decisions within households in rural Tanzania. Using original data from husbands and wives (interviewed separately) in 1,851 Tanzanian households, the analysis examines differences in the wife’s authority over 13 household and farming decisions. The study finds that the level of decision-making authority allocated to wives by their husbands, and the authority allocated by wives to themselves, both vary significantly across households. In addition to commonly considered assets such as women’s age and education, in rural agricultural households women’s health and labor activities also appear to matter for perceptions of authority. We also find husbands and wives interviewed separately frequently disagree with each other over who holds authority over key farming, family, and livelihood decisions. Further, the results of OLS and logistic regression suggest that even after controlling for various individual, household, and regional characteristics, husband and wife claims to decision-making authority continue to vary systematically by decision—suggesting that decision characteristics themselves also matter. The absence of spousal agreement over the allocation of authority (i.e., a lack of “intra-household accord”) over different farm and household decisions is problematic for interventions seeking to use survey data to develop and inform strategies for reducing gender inequalities or empowering women in rural agricultural households. Findings provide policy and program insights into when studies interviewing only a single spouse or considering only a single decision may inaccurately characterize intra-household decision-making dynamics.

## Highlights

• We use surveys of husbands and wives in Tanzania to explore rural household decision-making.
• We find perceptions of household decision-making authority differ depending on the spouse asked.
• Factors associated with a wife’s authority include age, education, health, children, and labor hours.
• The allocation of intra-household authority also varies across thirteen different decision questions.
• A lack of “intra-household accord” over authority may be a barrier to empowerment efforts.

## 1 Introduction

Except for the rare couple that shares common preferences and equal access to resources and information, the distribution of decision-making authority between spouses can be expected to affect the allocation of household resources.^1^ Scholars seeking to understand these intra-household dynamics have generated a rich literature on the broader measures, determinants, and household consequences of spousal bargaining power and decision-making (Doss, 2013, Kebede et al., 2013, Malapit and Quisumbing, 2014). These household dynamics are important to resource allocation in many contexts, and certainly in low resource, high risk, and relatively isolated environments with strong gender norms, such as arise in many rural parts of the developing world.

Since the 1980s the concept of women’s status has expanded from solely encompassing education and socio-economic levels to explicitly including women’s access to and control over resources and most recently, empowerment (Kabeer, 1999, Malhotra and Schuler, 2005, Mason, 1986, Mason, 2005, Mosedale, 2005).^2^ Although definitions vary, to be “empowered” is to have the rights, capacity, and assets to be able to make choices (Ibrahim & Alkire, 2007; and see Alkire, Meinzen-Dick, Peterman, Quisumbing, and Seymour (2013) for a fuller discussion). Intra-household bargaining power is one component of empowerment, for which decision-making authority is used as an indicator (Doss, 2013, Heckert and Fabric, 2013, Kabeer, 2001, Mason, 2005). We refer the reader to recent excellent reviews in Kebede et al., 2013, Doss, 2013, and Malapit and Quisumbing (2014) for a more thorough discussion of intra-household bargaining and decision-making processes. Our own focus is on the measurement of women’s bargaining power, including how indicators of women’s intra-household authority vary across husband and wife self-reports, and across multiple household decisions, particularly as such variation might inform policy and development interventions in a rural developing country context.

Understanding how farm households allocate intra-household authority is difficult given the paucity of data on key rural household decisions and decision-making processes. In this paper we use original data that show how perceived authority over multiple household and farm management decisions in rural Tanzania varies by spouse. In so doing we contribute to the literature on intra-household decision-making in two ways. First, while the existing literature mostly tests decision-making authority within a particular domain of decisions as a function of spousal characteristics such as age and education, our data cover 13 different farm household decisions, and include important farm household co-variates such as health and the division of labor among market, farm, and home. This allows us to examine the role of decision-maker characteristics across multiple decisions for the same household. Second, while existing empirical work is largely restricted to a single spouse’s account of decision-making authority and assumes the reported division of authority is understood by both spouses, our household survey is based on a relatively large random sample of farm households, including 1,851 complete husband-wife surveys, that asks the same questions of both spouses separately. This allows us to examine both husband and wife claims to decision-making authority and the incidence of accord and discord over those claims. In cases of discord, we posit that policy and development interventions may be misplaced if they are targeted based on analyses using decision-making reports from a single spouse.

The paper is structured as follows. In Section 2 we briefly review the literature on intra-household decision-making. Though our work is not designed to add to debates on the outcomes of decision-making authority, this literature provides the foundation for our empirical work, particularly studies focused on the determinants of decision-making authority and challenges to the unitary household model. Section 3 describes our data and methods, and we present our original findings in Section 4, using survey responses from a random sample of couples in Tanzania across 13 different agricultural and household decisions. We find a husband’s allocation of decision-making authority to his wife varies according to his wife’s age and education, consistent with hypotheses that having greater assets (in terms of human or physical capital) can offer better exit options for women and thereby increase their decision-making power. Novel, however, is our finding that for rural women for whom farming is the main livelihood, health status also matters, as does the relative amount of time the husband contributes to home labor. Findings also suggest that, on average, intra-household accord over which spouse holds decision-making authority is more likely in households where women have higher levels of education. But accord is lower in households where the woman is more active in market labor—in such cases the wife’s higher self-perception of decision-making authority is not matched by the husband’s perception (contrary to some findings for urban settings; e.g., Bertocchi, Brunetti, and Torricelli (2014) suggest market access increases women’s household authority from the perspective of both spouses). Overall our empirical investigation suggests that analyses based on population-level male and female averages may mask significant husband-wife differences of opinion over who holds decision-making authority within households. In cases of husband-wife discord, different development efforts to increase women’s authority and ultimate empowerment might be recommended depending on whether the wife or the husband is interviewed. Section 5 concludes.

## 2 Intra-household bargaining and decision-making authority

The relationship between intra-household decision-making authority, resource allocations, and positive outcomes for women and children has been observed in many different cultural and economic contexts. In India, for example, increased women’s authority relative to their husbands’ is associated with increased use of modern contraception and to declines in infant and child mortality (Jejeebhoy, 2002). Similar reproductive, maternal, neo-natal, and child health outcomes have been observed in Latin America (Becker, Fonseca-Becker, & Schenck-Yglesias, 2006), in Africa (e.g., in Egypt (Kishor, 2000) and Mali (Castle, 1993)), and in Southeast Asia (Beegle, Frankenberg, & Thomas, 2001). Increasing women’s bargaining power is associated with increased expenditure shares on key household goods such as health and education, which can lead to improved child outcomes (see Doepke and Tertilt (2014) for a comprehensive review). There has also been a recent and robust examination of nutritional outcomes relating to women’s household authority across multiple countries (Haddad et al., 1996, Richards et al., 2013), including Bangladesh (Bhagowalia, Menon, Quisumbing, & Soundararajan, 2012), Senegal (Lépine & Strobl, 2013), Nepal (Malapit, Kadiyala, Quisumbing, Cunningham, & Tyagi, 2013), and Ghana (Malapit & Quisumbing, 2014).

Given such evidence of the potential benefits of greater women’s intra-household authority, a growing body of empirical work in development economics has sought to identify predictors of women’s bargaining power in the household. Historically the simplest models of household decision-making have relied upon a unitary household model (Bobonis, 2009, Quisumbing and Maluccio, 2003). Such models effectively assume that household members pool household income and/or that husband and wife preferences can be treated as homogeneous (or, alternatively, that only the husband’s preferences are relevant determinants of household resource allocations). A vast body of scholarship, however, now suggests that husbands’ and wives’ relative intra-household decision-making authority is highly relevant to resource allocation, that is, most households do not fully pool income and in many cases spousal preferences are not homogeneous (Attanasio and Lechene, 2002, Duflo, 2003, Duflo and Udry, 2004, Haddad et al., 1997, Hoddinott and Haddad, 1995, Lundberg et al., 1997, Balasubramanian, 2013, Richards et al., 2013).

Our analysis draws from theoretical insights offered by various co-operative and non-co-operative bargaining models which offer alternative characterizations of intra-household decision-making processes that may better reflect actual patterns of decision-making than a unitary household model. Co-operative models posit that household bargaining outcomes are negotiated directly between spouses and that outcomes rely on each spouse’s relative ability to claim power and to threaten defection from a less-than-desired negotiation outcome by invoking an outside option, such as the threat of spousal sanctions through divorce (Manser and Brown, 1980, McElroy and Horney, 1981) or non-co-operation within marriage (Chen and Woolley, 2001, Kanbur and Haddad, 1994, Konrad and Lommerud, 2000, Lundberg and Pollak, 1993). Non-co-operative models assume independent actions on the part of both spouses lead to a self-enforcing Nash equilibrium, which may or may not be Pareto efficient (Lundberg & Pollack, 1994).^3^ One key difference in co-operative and non-co-operative models is the stability of the bargaining outcome: co-operative models are presumed stable in the absence of any changes to the spouses’ relative bargaining power, while non-co-operative equilibria may shift as new information about the spouse’s position and strength becomes available. Results consistent with non-co-operative bargaining models have now been observed across a range of developing country contexts (e.g., Katz, 1995, Kebede et al., 2013, Mabsout and Staveren, 2010, Malapit and Quisumbing, 2014, McPeak and Doss, 2006, Udry, 1996, Castilla and Walker, 2013), emphasizing the potential for shifts in women’s decision-making authority to lead to shifts in welfare and other outcomes for women and households.

In this context, some empirical research has focused on finding valid measures of decision-making authority in addition to measuring outcomes of women’s bargaining power (Agarwal, 1997, Basu, 2006). Most models consider women’s property, financial assets, and engagement in market labor to be key determinants of women’s authority over household decisions (Antman, 2014, Attanasio and Lechene, 2002, Bertocchi et al., 2014, Doss, 2006, Doss, 2013, Doss et al., 2014, Quisumbing, 2003, Quisumbing and Maluccio, 2003). Other factors such as age, education, and social and political assets (Chattopadhyay and Duflo, 2004, Duflo and Udry, 2004), spousal communication (Ashraf, 2009), trust and spousal contributions to the household (Iversen, Jackson, Kebede, Munro, & Verschoor, 2006), and institutionally determined gender norms and ideology (Bradshaw, 2013, Mabsout and Staveren, 2010) have also been examined. Some of these factors are predicted to affect the bargaining process while others may affect relative power via provisioning women with outside (exit) options (Rubalcava & Thomas, 2000). Recently, household composition (e.g., presence of children) has also been hypothesized to shape women’s decision-making roles (Gummerson & Schneider, 2013) and arguably also their exit options.

Though we have learned a great deal from this literature, asset-based models of intra-household decision-making leave open several unanswered questions, often driven by data constraints. First, studies are usually limited to a single or few household decisions. But real-life households engage in countless decisions, and simple asset-based models of spousal negotiating power cannot explain situations where the allocation of decision-making authority within a single household varies depending upon what decision is at stake. For example, recent findings by Bayudan-Dacuycuy (2013) suggest that the presence of a spouse’s parents in a household may strengthen wives’ bargaining power in the Philippines, but the effects are different for daily household decisions *versus* core household financial decisions. Such findings suggest previous studies predicting spousal decision-making authority across a single decision may have missed meaningful variation in spousal authority across different decisions.

Second, most studies of intra-household decision-making consider only a single spouse’s report of relative authority. But data from a single spouse ignores the possibility that husbands or wives may not agree with the other spouse’s assessment of household decision-making authority. This lack of information on spouses’ relative dispositions toward household decisions may be an especially serious weakness of past studies, since household outcomes ultimately depend on the behavior of two (or more) individuals who may agree or disagree on any specific course of action. Disagreement over decision-making authority may particularly affect women, and if the preponderance of survey respondents are male heads of households, then gaps in our understanding of women’s true authority may be particularly acute. Anecdotal evidence from our fieldwork suggests many husbands report that decision-making within the household is shared, but when spouses are asked if this is true, the wives strongly disagree. Understanding gaps in women’s perceived *versus* actual decision-making authority may help explain some adoption paradoxes in programs targeting women. For example, Miller and Mobarak (2013) find “that women–who bear disproportionate cooking costs–have stronger preferences for healthier stoves, but lack the authority to make purchases. Our findings suggest that if women cannot make independent choices about household resource use, public policy may not be able to exploit gender differences in preferences to promote technology adoption absent broader social change.”

The available evidence suggests that discrepancies between husband and wife reports of household matters may be large: in a study of couples in Tamil Nadu and Uttar Pradesh, India for example, Jejeebhoy, 2000, Jejeebhoy, 2002 finds husbands and wives differ widely in assessments of the woman’s level of mobility, her access to economic resources, and her decision-making authority. Indeed, for the wife’s involvement in the purchase of food, major household items, and jewellery, the spouses gave inconsistent reports in as many as 50% of couples. Ghuman, Lee, and Smith (2004) analyze similar survey data from India, Pakistan, Thailand, the Philippines, and Malaysia and conclude that men and women not only differ in their assessments of women’s decision-making authority, but in some cases even have different understandings of the questions, differentiating between “having final say” and “having input” in very different ways. Bradshaw (2013) found men and women in Nicaragua differ significantly in their estimates of women’s household labor contributions, particularly in rural areas where men dramatically under-value women’s income-generating activities relative to women’s own self-reports.

Intra-household discord, or husband-wife discrepancies in self-reported authority over household decisions, has two potential implications. First, from a household resource perspective discordant couples may suffer from inefficiencies in individual and household resource use–if both spouses assume they have decision-making authority and preferences differ, efforts could be either duplicative (both spouses do the same work) or decision-related activities may be neglected (if each spouse perceives the other as responsible). Second, the presence of intra-household discord may have important policy implications; namely, for a given decision if husbands and wives both claim authority, or both defer authority, the results of interviewing one or the other spouse about household decision-making processes (important, among other things, for targeting development interventions) may lead to erroneous conclusions. If the biases present in the results of surveys that only interview one spouse are random, little is to be gained from worrying about discord. If the biases are not random, however, then we can potentially learn how to better target efforts involving intra-household decision-making.

## 3 Description of the sample and measures of intra-household decision-making and accord

Our empirical strategy is to take advantage of a new and unusually rich dataset to inform key outstanding questions around intra-household decision-making authority. Our first set of estimations begins with a single spouse’s responses: the husband’s attribution of authority to his wife, denoted as W*_i_*[H*_i_*]. We test if, and in what manner, our conclusions about the allocation of intra-household authority differ depending on which spouse is asked, by considering both husbands’ reports of the wife’s authority W*_i_*[H*_i_*] and wife reports of her own authority W*_i_*[W*_i_*].^4^ We additionally explore whether authority differences vary across decisions, possibly suggesting authority derives from characteristics of the decisions in addition to the more studied spousal characteristics such as age, education, and financial assets. Our second set of estimations focuses on spousal accord—a measure of the degree to which the husband and wife in household *i* agree on relative decision-making authority—to look at those decisions where spousal perspective does matter (that is, where W*_i_*[H*_i_*] − W*_i_*[W*_i_*] ≠ 0), and for which conclusions and policy recommendations would hence differ depending on which spouse was consulted.

Data were collected in October, 2010 by the East African office of TNS Research International. Respondents are a random national sample of 1,851 Tanzanian rural smallholder households, with “smallholder” defined locally though generally restricted to households farming less than 20 acres of primary crops including maize, beans, cassava, and rice.^5^ Local enumerators used a probability proportional to size sampling approach with the ward (the smallest administrative unit in Tanzania) as the sampling point. Working from the national population census for Tanzania and eliminating Zanzibar and urban sampling points, approximately 300 wards were distributed across 102 districts in proportion to population size. Within wards, households were selected using the random walk technique. Because the survey was restricted to mainland rural smallholders and our sub-sample is only households with a married male head (excluding female-headed households or households in which another male, such as a relative, has assumed the role of head-of-household), our sample is not nationally representative. The survey questionnaire was pre-tested locally several times and gathered information on household characteristics including demographics of all household members and detailed information on farming practices.

A key objective of the survey was to identify psychographic characteristics of farmers in order to develop agricultural interventions that could target farmers based on their underlying motivations and attitudes toward farming. The male and female primary decision-makers were defined for enumerators as “the adult person who is responsible for the organization and care of the household or who is regarded as such by the members of the household.” The male head of household answered all questions in the survey, while both spouses separately answered a subset of questions that included personal information, attitudes toward farming and risk, and perceptions of decision-making authority. To reduce response bias, especially originating from cultural norms or expectations, spouses were interviewed separately, and individually.

The reported allocation of household decision-making authority is assessed through 13 questions about household and farm activity areas. Each respondent was asked to allocate 10 beans between the husband and wife to reflect each individual’s relative decision-making authority over a given decision. Questions were worded as follows:

Thinking of yourself and your spouse, how is household decision-making shared between the two? I am giving you 10 beans and I want you to share them between yourself and your spouse according to the power each has in making the decision.

### (a) Farm and cash decisions

• What crops to cultivate in the farm?
• Where to sell cash crops?
• When to sell off livestock?
• How to spend cash from the sale of cash crops?
• How to spend cash from the sale of livestock?

### (b) Decisions about children

• What foods to feed the family?
• Whether to send children to school?

### (c) Innovation decisions

• Whether to buy a new high-yield seed variety or use the ordinary seeds?
• Whether to buy new farm equipment or stay with the old tools?
• What types of information or training the household needs?
• Who to attend farm training?

### (d) Broad decision-making authority: livelihood *versus* overall

• What happens in the farm generally?
• Overall decision-making for the household?

Husbands and wives were interviewed separately to ensure spouses did not influence each other’s allocations. Each respondent was also required to allocate all 10 beans between him or herself and the spouse. For example, if a husband assigned himself 7 beans for authority over what happens on the farm generally, he had to assign the remaining 3 beans to his wife. Third-party decision-makers, such as in-laws, brothers and so forth, were not eligible for consideration. Alternately, the respondent could state that the decision was not applicable to their household at all (for example for households with no livestock certain livestock-related decisions might not apply). This resulted in a code of N/A for both husband and wife decision-making authority.

#### (i) Comparing spousal reports of decision-making authority

We first explore determinants of men’s reported allocation of authority to their wives W*_i_*[H*_i_*] across the thirteen decisions. Reported authority is measured on a scale from 0–10, according to the number of beans allocated by each spouse to themselves and their spouse. We consider four groups of variables: wife-specific characteristics and assets; household and farm shared assets; husband/wife attribute and asset differences; and regional effects that capture some ethnic differences. Descriptive statistics for key explanatory variables are summarized in Table 1.

*Wife-specific characteristics and assets* are measured by the wife’s age, years of education, health (1 = very poor to 5 = very good), and the number of labor hours she reports spending working on the farm, in the market, and at home in a typical day.

*Household shared assets* include an overall asset score computed as a 100-point scale assigning weights to different assets owned by the household, including the wall material of the respondent’s home, the floor material of the best room in the house, the main roofing material, a description of the number of rooms and household furniture, household sanitation facilities, agricultural assets, cooking implements, power and lighting, and other household items. In addition, we use a proxy for household community standing, in which the male head of household places himself on a ten-rung ladder representing his relative standing in the community. We further consider other theoretically important household attributes including total acres of land cultivated, the distance to a paved road, and whether or not the household has any children under age ten.

*Regional effects* are captured through dummy variables for seven administrative regions: Eastern, Central, Southern Highlands, Lake Northern, Southern, and Western (with Eastern omitted from the regression models). These are included to capture variation arising from unmeasured differences in social norms (especially to the extent that they reflect religious and ethnic group clusters potentially impacting women’s authority (Mabsout & Staveren, 2010)), as well as variation in farming practices, exposure to foreign programs and communities, etc. Estimates from the Tanzania Demographic and Health Survey (TDHS) suggest considerable variation among regions across several key indicators including measures of empowerment such as the wife’s participation in decisions (defined as the sole or joint decision-maker) in the woman’s own health care, in major household purchases, and in visiting family, relatives and friends (Tanzania Demographic and Health Survey (TDHS), 2010).The resulting ordinary least squares (OLS) regression for the wife’s authority as reported by the husband in household *i* and in region *j* takes the form:

(1) $$W_{\mathrm{ij}}[H_{\mathrm{ij}}]=\beta_{0}+\beta_{1}{\text{WIFE}}_{\mathrm{ij}}+\beta_{2}{\mathrm{HOUSEHOLD}}_{\mathrm{ij}}+\beta_{3}{\mathrm{SPOUSE}}_{\mathrm{ij}}+\beta_{4}{\mathrm{ZONE}}_{j}+\varepsilon_{\mathrm{ij}}$$

where WIFE is a vector of wife-specific characteristics in household *i*, HOUSEHOLD is a vector of household assets (shared by the wife and her husband), SPOUSE is a vector of husband assets relative to the wife represented as the difference in wife and husband responses to individual asset-related questions within a given household, and ZONE is a series of dummy variables controlling for regional effects associated with each Tanzanian zone *j*.

### (ii) Intra-household accord over decision-making authority

We then test whether aggregate male/female agreement on the share of decision-making authority accurately represents husband/wife accord (measured by the difference in wives’ and husbands’ reports of women’s decision-making authority, ranging from -10 to + 10 beans for each question). To analyze accord by decision, we use the difference between the wife’s authority as reported by the husband and the wife’s self-reported authority, or W_i_[H_i_] − W_i_[W_i_].^6^ We next use logistic regression to predict the probability of accord for each household decision controlling for demographic covariates available from the survey data and hypothesized to be associated with intra-household decision-making and accord.

An initial logistic equation was specified at the household level for each decision as:

(2) $$\begin{array}{cc} \;\text{Where}: & \\ \left(\begin{array}{cc} \text{If}|{\text{W}}_{i}[{\text{H}}_{i}]-{\text{W}}_{i}[{\text{W}}_{i}]|<2\text{,} & {\text{Accord}}_{i}=1\\ \text{Else} & {\text{Accord}}_{i}=0 \end{array}\right\} & \end{array}$$

(3) $$\text{Log}[\text{P}({\text{Accord}}_{\mathrm{ij}})/(1-\text{P}({\text{Accord}}_{\mathrm{ij}})]=\beta_{0}+\beta_{1}{\text{WIFE}}_{\mathrm{ij}}+\beta_{2}{\text{HOUSEHOLD}}_{\mathrm{ij}}+\beta_{3}{\text{SPOUSE}}_{\mathrm{ij}}+\beta_{4}{\text{ZONE}}_{j}+u_{\mathrm{ij}}$$

Variable definitions are identical to those in Eq. (1). Coefficients on all variables reflect the increase in the log odds likelihood that the household with the applicable characteristic will be in accord (|W*_i_*[H*_i_*] − W*_i_*[W*_i_*]| < 2) for a given decision.

In the final analysis we restructure the data in long form such that each decision within a household is assigned the characteristics of that household, resulting in a household-decision dataset where each household appears 13 times, once for each decision question asked of that household. With this long-form dataset the final regression models are represented as:

(4) $$\begin{array}{c} \text{Log}[\text{P}({\text{Accord}}_{\mathrm{ijk}})/(1-\text{P}({\text{Accord}}_{\mathrm{ijk}})]=\beta_{0}+\beta_{1}{\text{WIFE}}_{\mathrm{ijk}}+\beta_{2}{\text{HOUSEHOLD}}_{\mathrm{ijk}}+\\ \beta_{3}{\text{SPOUSE}}_{\mathrm{ijk}}+\beta_{5}{\text{ZONE}}_{\mathrm{jk}}+\beta_{6}{\text{DECISION}}_{k}+v_{\mathrm{ijk}} \end{array}$$

After clustering by zone and question, this data structure allows us to systematically test the effects of each decision *k* on the probability of accord, controlling for household characteristics.

## 4 Results

Our first set of estimations adds to earlier findings on decision-making authority as seen from the perspective of one spouse, but looks across multiple decisions for the same household. Additionally, we examine whether which spouse is surveyed matters, as evidenced by different perceptions of authority by spouses over different decisions.

### (i) Predicting wife’s authority as a function of wife and household characteristics

Table 2 and Table 3 show the results of OLS regression models predicting the authority (number of beans out of 10 possible) allocated to wives by their husbands (W*_i_*[H*_i_*], Table 2) and to husbands by their wives (H_*i*_[W*_i_*], Table 3) across 13 farm and household decisions, as a function of the wife’s assets and household characteristics. These results are summarized in Table 4, where the shading of each cell indicates whether the significance and sign for the estimates for the husband allocating beans to his wife is different (light or dark gray) or the same (white or black) as the estimates in the same regression run on the wife’s allocation of beans to herself. Cells shaded in light gray are significant predictors of the number of beans allocated to the wife by her husband (W_i_[H_i_]). Cells shaded in dark gray are significant predictors of the number of beans that the wife allocates to herself (W_i_[W_i_]) (these model results are not shown, but are available in the data Appendix). White (unshaded) cells are not significant in either estimation. Finally, cells shaded in black are significant predictors of the allocation of authority in both husband and wife allocations of intra-household authority.

Variables significantly associated with the wife’s authority over farm and household decisions as perceived by both husband and wife include the wife’s age (higher authority), the number of hours that the wife works on the farm (higher authority), the husband’s community standing (lower authority), the difference in the number of hours worked in the home between the husband and wife (higher authority as the husband works more at home or the wife works less at home) and the zone where the household is located (relative to the eastern zone, wives on average in the southern zone have more authority; the effect of other zonal variables varies by decision). The wife’s authority over farm decisions and cash (Models (1) to (5)) is most associated with wife-specific attributes and assets such as her age and education (both increasing the wife’s authority over crop and cash decisions in the eyes of both husband and wife). In contrast, authority over innovation decisions (Models (8) to (11)) such as the purchase of new seed or farm tools is more strongly associated with household and spousal characteristics including household assets (with more assets generally decreasing the wife’s authority as reported by the husband, but in the case of seed purchases increasing the wife’s self-reported authority) and differences in the husband’s and wife’s health and labor activities (the wife having less authority as the husband becomes relatively healthier and/or more active in farm labor and less active in home labor).

For decisions about children (Models (6) and (7)) and general farm (Model (12)) and household (Model (13)) authority, a diverse combination of wife-specific, household-specific, spousal and regional variables all appear to play a part, with some interesting findings. For example, the finding that increasing distance to a paved road is associated with more authority allocated to wives over key marketing and food decisions may reflect fewer market and food options in rural areas, thus rendering the question of “who decides?” less important (resulting in a more even sharing of reported authority between spouses). The finding that higher community standing as reported by the husband is associated with lower authority for the wife over cash, seed purchases, and general farm and household decision-making may be driven by a husband’s self-reported standing in the community being similar to his self-assessment of the allocation of authority he holds within his household.

There are also noteworthy differences in predictors of the wife’s authority depending on who is asked (the husband or the wife) and on the decision itself. In terms of spousal responses, the husband’s perception of the wife’s authority (in light gray in Table 4) for several questions was additionally influenced by the wife’s health, home labor, distance to a paved road and the presence of children in the household. The wife’s description of her own authority (in dark gray) meanwhile was more strongly related to her education, health, farm size, and farm and market labor participation (especially relative to her husband). On average, higher participation in farm labor is associated with women allocating themselves higher authority for cropping decisions, children’s schooling, and training, but appears to constrain their self-reported authority over purchases of inputs. Higher participation in market labor is associated with higher wife authority over crop sales decisions (as reported by both wives and husbands), but lower overall authority in the household (as reported by wives, but not by husbands). The absolute number of hours that the wife works in the home is only significant in the husband’s allocation of authority to his wife; it is not a significant predictor of the wife’s perception of her own authority.

Another striking finding is that the presence of young children in the household—though associated with a relatively large increase in a wife’s overall authority as allocated by her husband (Model (13))—is also associated with a lower wife authority for several specific decisions, including decisions about what crops to grow and where to sell them (Models (1) and (2)) and whether to invest in assets and training (Models (9) to (11)). Surprisingly, the presence of young children in the household is also associated with lower wife authority as allocated by the husband for decisions on what foods to feed the family and children’s schooling (Models (7) and (8)). In the latter case, women also allocate less authority to themselves around these decisions, perhaps suggesting that the presence of young children constrains women’s active participation in household decision-making. Alternately, this pattern may again suggest that husbands and wives in our sample were more likely to assign higher authority to wives over less important (or more abstract) decisions, such as family nutrition and children’s schooling decisions in households with no young children.

Lastly, differences in age or education level between husbands and wives are not generally associated with allocations of authority, but perhaps not surprisingly for farm households, health differences, and differences in labor input are. On average, men rate themselves about half a point higher than women in health on a 5-point scale. The regression coefficients in Table 2 thus suggest that as husbands rate themselves as increasingly healthy relative to their wives, their allocation of authority to wives decreases across a range of decisions. Moreover, both men and women show this tendency for several key decisions, suggesting that relative health status may be a strong predictor of household authority. Farm labor differences also matter: on average men allocate 0.6 hours more per day to farm labor than their wives, but as a wife’s relative farm labor input rises (perhaps implying a lack of alternative income sources), the husband’s allocation of authority to her, especially around innovation decisions, decreases. Differences in home labor also affect allocations of authority. The average home labor difference is strongly negative (with the wife contributing more home labor than the husband), thus the coefficient estimates suggest that a positive change in the difference in home labor (i.e., as this difference becomes a smaller negative number, with the husband contributing more to home labor relative to the wife, or the wife contributing less relative to the husband) the wife’s authority increases for most decisions. In other words, more equal home labor input appears to be associated with higher decision-making authority for women–although we cannot tell if more equitable spousal labor allocations are the cause of, or the result of, relative authority.

Together the above models indicate, consistent with the published literature on intra-household negotiation, that women’s authority within the household is shaped by her characteristics and assets (both absolutely and relative to her husband), as well as by household-level attributes such as overall socioeconomic status and regional characteristics such as variation in farming systems and traditional practices. Moreover these models also provide strong evidence that the processes by which husbands allocate authority to their wives and the processes by which wives allocate authority to themselves differ depending upon both the characteristics of the husbands and wives and upon the characteristics of the questions being asked.

### (ii) Allocation of authority within households: accord between husbands and wives

Our subsequent set of estimations uses spousal accord–a measure of the degree to which the husband’s allocation of authority to the wife (W*_i_*[H*_i_*]) and the wife’s allocation of authority to herself (W*_i_*[W*_i_*]) reflect intra-household agreement on relative decision-making authority–to highlight decisions where spousal perspective matters for intra-household decision-making dynamics.

**Figure 1:**
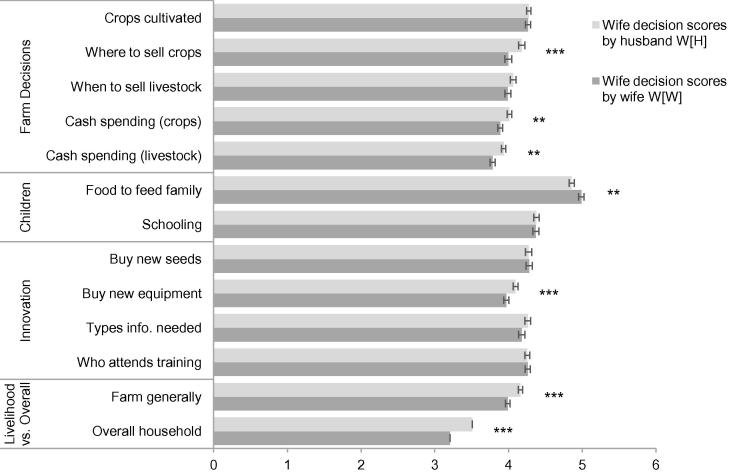
Beans allocated to wives, averages by husbands W*_i_*[H*_i_*] and by wives W*_i_*[W*_i_*] by decision Mean out of 10 beans, with standard errors and significant differences using unpaired *t*-tests. ^∗^*p* < 0.10; ^∗∗^*p* < 0.05; ^∗∗∗^*p* < 0.01.

Figure 1 summarizes the average amount of decision-making authority attributed to women as reported by men for each of the 13 questions in our data. With the exception of what foods to feed the family, on average nearly all of the decision-making variables appear to fall predominantly in the domain of the husband (that is, out of 10 beans less than 5, and often less than 4, beans on average are attributed to wives). At first glance men’s and women’s reports of decision-making authority appear surprisingly consistent–the difference in average reports by men and the average reports by women rarely approaches even one fifth of one bean, with mean differences for men’s and women’s reports on six out of the 13 questions not statistically different from zero.

A few things stand out from these averages. There is little evidence of women substantially under-reporting their decision-making authority relative to men’s reports. Women’s under-reporting of their own authority is a pattern that has been noted in numerous past quantitative and qualitative studies (Becker et al., 2006). More consistent with previous research, Figure 1 highlights one decision where women consistently have relatively more authority than other decisions–what foods to feed the family. Even for this decision, however, women do not have more authority than men, and at most are perceived to share it equally (mean W*_i_*[H*_i_*] = 4.86; mean W*_i_*[W*_i_*] = 4.99).

When we examine the allocations of authority across spouses, however, the results look quite different. Namely, aggregate responses across men and women appear to mask substantial household-level differences between the reports of husbands and wives–producing misleading conclusions about the general level of intra-household accord in a population. To capture variation in husband and wife responses to individual decision-making questions we define the level of accord for a given decision as: the level of wife authority reported by her husband W*_i_*[H*_i_*] minus the level of wife authority reported by the wife herself W*_i_*[W*_i_*]. Denoted as W*_i_*[H*_i_*] − W*_i_*[W*_i_*] this household decision-level accord variable ranges from + 10 if a husband in household *i* thought a particular decision was entirely in the wife’s domain and the wife thought it was entirely in the husband’s domain (potentially reflecting neglect or indecision on the issue), to 0 if the husband and wife agreed on who held decision-making authority, to -10 if both husband and wife thought the decision was in their own domain alone (reflecting potential conflict over decision-making authority). Note that since all spouses were given 10 beans to allocate, it does not matter whether we use the number of beans allocated to husbands or wives–the difference is identical.

**Figure 2:**
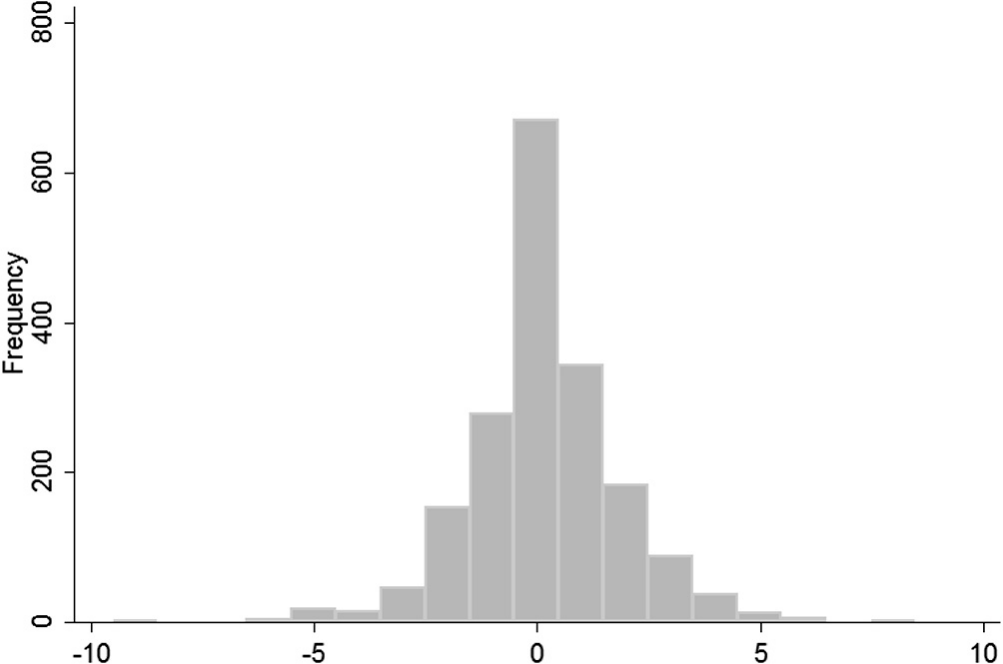
Intra-household accord (W*_i_*[H*_i_*] − W*_i_*[W*_i_*]) on authority over “farm decisions generally”.

As illustrated in Figure 2, there was a great deal of variation in the distribution of intra-household accord for the different decisions, including on the question of general farm decision-making authority.

As further shown in Table 5, looking across all of the decisions, such husband/wife disagreements are widespread. Differences in perceptions range up to a seven bean or more on all questions, and mean accord scores are significantly different from zero for nine out of the 13 decisions.

The most significant discord in reported decision-making authority was for broad areas of household activity (for example responsibility for general farm decisions, or for decisions in the household overall) and for cash-related questions, such as cropping, sales, and equipment purchase decisions. Almost all significant relationships involve husbands reporting more authority for their wives than wives report for themselves (implying possible neglect of these decisions, with both husbands and wives attributing more authority to the other than the other attributes to him or herself). Interestingly, the decision on what foods to feed the family was the only one of the thirteen decisions for which a hypothesis that the difference between husbands and wives was less than zero could not be rejected (t = −2.604; p < 0.005), implying both husbands and wives claimed more authority than was acknowledged by the spouse.

### (iii) Predicting accord as a function of household characteristics

To simplify the presentation of results, the ordinal accord variables were transformed into dichotomous variables, with households classified as either in accord (with spousal allocations of authority reflecting less than a difference of two beans) (|W*_i_*[H*_i_*] − W*_i_*[W*_i_*]| <2) or not in accord (with the difference in spousal allocation of authority of +2 beans or greater, or −2 beans or less.

Table 6 reports the marginal effects from logistic regression for all questions with this binary dependent variable. Given the cross-sectional nature of our data, these covariates should be interpreted as the association between spousal and household characteristics and accord, since we are unable to deal with possible endogeneity. We again report independent variables in four categories: wife specific assets (age, education, health, and labor); household assets and attributes (shared household assets, landholdings, distance from road, and the presence of children under 10); spousal differences (differences in age, education, health, and labor); and regional effects.

Among significant predictors of accord, wife-specific characteristics are particularly important. Self-reports of decision-making authority by older, more educated wives more closely match their husband’s reports. The effect of wife health on accord depends upon the decision: relatively healthier wives are more likely to agree with their husbands on the allocation of authority over schooling decisions (Model (7)), but more likely to disagree with their husbands over who holds authority for farm decisions in general (Model (12)). More hours of home labor for the wife is also associated with less accord between her and her husband over the allocation of decision-making authority over several farming and household decisions (Models (3), (4), (8), and (13)).

Household shared assets and attributes, as a group, were the variables most strongly and consistently associated with the probability of accord. Having more assets and a higher community standing (as reported by the husband) both decrease the probability of accord over several different farming, innovation, and general farm/household decisions. Inversely, being further from a paved road was almost universally associated with an increase in the probability of accord. Together these results suggest that more marginal households are likely to have greater accord, all else being equal. It is unclear whether these results derive from lower costs of negotiating authority in households that simply have fewer options, or whether households with fewer decision options are a result of accord–recognizing that accord can derive from autocratic husbands and abstaining wives adhering to shared gender norms.

Age and educational differences between husbands and wives were relatively weak predictors of accord for most decisions, however the relative health of wives *versus* their husbands is a significant predictor of accord over some key farming decisions, with less healthy wives less likely to agree with their healthier husbands.

Finally, among regional effects, relative to the Eastern zone, the Central and Southern zones tend to have lower accord across most farm and market decisions, while the Southern Highlands and Western Zone tend to have a higher incidence of accord, particularly for decisions involving innovation and general/overall farm and household authority. The only decision for which there is no significant regional variation in accord is for who holds authority over children’s schooling (Model (7)).

Together the models in Table 6 clearly show that the relative significance and importance of spousal and household characteristics varies by decision, thus implying that other factors, including characteristics of the decisions themselves, may affect the probability of accord.

### (iv) Predicting accord as a function of household and decision characteristics

Our final logistic regression model, as shown in Table 7, predicts the probability of intra-household accord as a function of both household and decision characteristics using a long-form household x decision dataset (*n* = 22,815 household x decision observations). The binary outcome variable is again the presence or absence of accord, with accord = 1 if the spousal allocation of authority for a given decision within a given household reflects less than a difference of two beans (|W*_i_*[H*_i_*] − W*_i_*[W*_i_*]| < 2), otherwise accord = 0, suggesting the lack of a common understanding of the division of authority within the household for that decision. The decision on “what crops to grow” is omitted from the model, such that the results for the other twelve decisions are relative to the likelihood of accord on this decision. These results support the earlier findings that the likelihood of accord varies by decision type, after controlling for household characteristics.

Looking across all 13 farm and household decisions simultaneously, more educated wives and wives with better health (relative to their husbands) are associated with a higher likelihood of accord over household decisions. Accord is also positively associated with more acres of landholdings and greater distance to paved roads. Conversely, in households where wives spend more time engaged in market labor accord is less likely. Wealthier households (as measured by assets) and households with a higher husband’s community standing are also less likely to be in accord.

As for the effects of decisions themselves on intra-household accord, controlling for household characteristics, accord over decision-making is relatively more likely for highly visible farm and household decisions including decisions on children’s schooling, general farm decisions, as well as for specialized decisions on whether to buy new seed or buy new tools (all not significantly different from the reference decision category, what crops to grow). All other decisions, especially those involving how to use cash from crops and livestock and who holds overall authority in the household, are associated with discord, even after controlling for wife characteristics, household assets, and spousal differences.

It is speculative exactly what the specific decision covariates are reflecting. Our results, however, appear consistent with the hypothesis that more privately appropriable benefits (such as cash decisions, or who attends trainings) as well as more difficult-to-monitor outcomes (such as what types of training are needed) may align with a greater probability of discord over the allocation of decision-making authority. More difficult-to-define decisions such as “overall household authority” (a phrase which each spouse might interpret quite differently) may also have a greater likelihood of discordant spousal reports.

## 5 Conclusions

We believe this research makes several contributions. Using original data from 1,851 Tanzanian households, we find that the intra-household allocation of decision-making authority cannot be explained by household demographic characteristics alone. That is, the rules of decision-making are not uniform across the board in a given household, but rather the allocation of authority to wives by husbands (and to husbands by wives) also varies across decisions within households. More novel, by exploiting responses by both husbands and wives, we further find that different individual and household-level factors explain variation in the perceived allocation of decision-making authority for husbands *versus* wives.

We also demonstrate that aggregate male–female agreement over the allocation of decision-making authority in a population can indeed overstate husband–wife accord over the allocation of authority within households. The average allocations of authority by men and by women in our sample are almost identical: generally differing by no more than one-fifth of one bean. In contrast, for husbands and wives, more than 65% of household spousal allocations differ by one bean or more, more than 30% differ by two beans or more, and there are significant differences in spousal reports for nine of the thirteen decisions considered in this study. Regardless of what is viewed as a “fair” distribution of decision-making authority, our prior belief based on male–female aggregates–that the prevailing distribution of authority over farm and household decisions is similarly understood between husbands and wives–has been challenged. Instead, our data indicate multiple cases where spouses disagree over who holds authority over different decisions, suggesting that valuable rights over decision-making authority remain incompletely defined.

These findings have important methodological implications: asking both women and men to independently report on their spouse’s decision-making authority entails significant increases in the costs and complexity of survey collection and analysis, and as a consequence many past studies have relied on assumptions that individual responses can be treated as equivalent to household responses. Our study has tested such assumptions empirically, using data from husbands and wives in households where men still hold more power, but where specialized or shared decision-making is relatively common. The results suggest widespread disagreement among husbands and wives in rural Tanzanian households, and the question of “who is in charge” hinges critically on who is asked and on the disposition of the spouse who may or may not agree.

These findings also have important policy implications. In addition to highlighting how research based on single-spouse interviews or a limited number of decisions may lead to erroneous conclusions surrounding intra-household decision-making dynamics, our results also suggest that investing in women as a means of increasing bargaining power may have differential pay-offs depending on the views of each spouse and on the decision being considered. Women’s education and women’s health, for example, appear to be positively associated with a wife’s allocation of overall household authority to herself, but not with corresponding allocations of overall authority by her husband. In contrast, higher women’s education *is* associated with shared spousal views of greater women’s authority over cash decisions, suggesting investments in education may improve women’s bargaining power, but unevenly so across different decision types. The effect of better women’s health, meanwhile, is associated with an increase (perceived by both husband and wife) in her authority over several key farm and household decisions, which ultimately suggests that, for agricultural populations in particular, one’s own physical capabilities are a crucial component of bargaining power.^7^

This result is reinforced by the significance of our labor findings: though there is variation across decisions, in general, more hours spent by a wife working on the farm are associated with more farm-related decision-making authority. The same result does not hold for hours the wife spends in market labor, suggesting that the effects of interventions to increase women’s participation in market labor in isolation (without increasing education or other factors related to intra-household negotiations) may not increase wives’ farm and household decision-making authority. Recognizing the value of collecting both husband and wife responses in research on important household resource allocation decisions can help advance methods for targeting agricultural development interventions to decision-makers (Doss, 2002, Duflo, 2011, FAO, 2011, Gilligan et al., 2014, Kilic et al., 2015, Malapit et al., 2014, Sraboni et al., 2014, van den Bold et al., 2013).

Based on bargaining theory and the results of this research we can develop some preliminary hypotheses about when and how differences in husband and wife views on authority might matter most. For example, we might expect the difficulty and duration of negotiations over authority to vary with the size and distribution of the expected benefits and costs of the outcome. Hence in general, we can expect more difficult negotiations (and likely more discord) over decisions involving greater payoffs, and more privately appropriable goods (for example cash-related decisions) relative to public household goods (for example nutrition choices, or investments in children’s education). The ability to monitor and enforce decision-making authority is another potentially important factor contributing to whether or not spouses have a common understanding of the division of authority. Infrequent and specialized decisions, such as an annual decision of where to sell crops or livestock as opposed to a daily decision of what foods to eat, limit the opportunities for spouses to assess their shared understanding, and might therefore be associated with greater discord. Cash-related decisions can be particularly difficult and costly to monitor, as a rich literature hypothesizes over seemingly inefficient behavior wives engage in to keep money out of the hands of male family members (e.g., Anderson and Baland, 2002, Schaner, 2011), further increasing the potential for discordant spousal views. In contrast, large durable purchases such as equipment tend to be more visible, and thus spouses might be expected to share a clearer understanding of how such decisions surrounding the use of household resources are made.

Research that collects data on the transaction costs associated with negotiating and monitoring the allocation of intra-household authority, along with sampling that includes all households (not just those with male heads) and identifies the decision-authority impact of particular investments in health, education, or labor market interventions, could, we believe, help test these hypotheses.

## Figures and Tables

**Table 1:** Summary of variables

Variable	Obs.	Mean	Std. Dev.	Min	Max
*Wife-specific characteristics and assets*					
Wife Age—in years	1851	37.407	10.846	18	80
Wife Education—in years	1851	1.883	0.657	1	7
Wife Health—personal rating from very good (5) to poor(1)	1851	3.987	0.784	1	5
Wife Farm Labor—hours in typical day	1850	4.233	1.971	0	12
Wife Market Labor—hours in typical day	1836	3.813	2.467	0	12
Wife Home Labor—hours in typical day	1851	4.926	2.491	0	12

*Household shared assets*					
HH Asset Index—100 point scale increasing in asset ownership	1851	23.579	8.774	0	80
Husband’s Community Standing—10 is highest	1851	4.470	1.602	1	10
HH Land Cultivated—in acres	1851	42.793	41.237	0	150
HH Distance to Pavement—in kilometers	1778	6.178	8.149	0.02	80
HH Children < Age 10 = 1 if any children < age 10	1851	0.621	0.485	0	1

*Spousal differences* (husband—wife)					
Age Difference—in years	1851	8.214	6.940	−25	44
Education Difference—in years	1826	0.146	0.727	−5	5
Health Difference—5 point scale	1851	0.061	0.971	−4	3
Farm Labor Difference—in hours	1851	0.590	2.536	−12	10
Market Labor Difference—in hours	1851	−0.473	2.896	−12	10
Home Labor Difference—in hours	1851	−3.308	2.835	−12	6

*Regional effects*					
Eastern	1851	0.145	0.352	0	1
Central	1851	0.034	0.181	0	1
Southern Highlands	1851	0.135	0.342	0	1
Lake	1851	0.128	0.334	0	1
Northern	1851	0.163	0.369	0	1
Southern	1851	0.205	0.404	0	1
Western	1851	0.185	0.389	0	1

**Table 2:** OLS results—number of beans allocated to wives by husbands (W_i_[H_i_]) by decision as a function of wife assets and relative spousal attributes

Models	(1)	(2)	(3)	(4)	(5)	(6)	(7)	(8)	(9)	(10)	(11)	(12)	(13)
	**Farm decisions and cash**					**Children**		**Innovation**				**Livelihood *versus* overall**	
	What crops to grow	Where to sell crops	When to sell livestock	How to use cash from crops	How to use cash from livestock	What foods to feed family	Children’s schooling	Whether to buy new seed	Whether to buy tools	What training needed	Who attends training	Farm generally	Overall authority
Wife Age	0.007⁎⁎	0.007⁎	0.012⁎⁎	0.010⁎⁎⁎	0.010⁎	0.007⁎	0.004	0.000	0.005	0.005	0.008⁎	0.010⁎⁎⁎	0.007⁎
Wife Education	0.012	0.010	0.012	0.034⁎⁎⁎	0.041⁎⁎	−0.005	0.015	0.013	0.017	0.026⁎	0.016	0.024⁎⁎	0.021
Wife Health	0.053	0.141⁎⁎	0.172⁎⁎	0.093	0.100	0.048	0.03	−0.027	0.163⁎⁎	0.166⁎⁎	0.268⁎⁎⁎	−0.015	0.107
Wife Farm Labor	0.049⁎⁎	−0.064⁎⁎	−0.016	−0.012	0.046	0.074⁎⁎⁎	0.053⁎	−0.002	−0.019	0.000	−0.052	−0.043⁎	0.048⁎
Wife Market Labor	0.008	0.018	−0.024	−0.043⁎	−0.015	0.045	−0.028	0.003	0.010	−0.026	−0.038	−0.004	−0.028
Wife Home Labor	0.025	0.055⁎	0.050	0.084⁎⁎⁎	0.032	−0.054⁎	0.078⁎⁎	0.050⁎	0.057⁎⁎	0.093⁎⁎⁎	0.047	0.093⁎⁎⁎	0.098⁎⁎⁎

HH Asset Index	−0.011⁎⁎⁎	0.000	0.002	0.000	−0.004	0.002	−0.008⁎	−0.008⁎⁎	0.000	−0.009⁎	−0.015⁎⁎⁎	−0.003	−0.008⁎
Husband’s Community Standing	0.024	−0.076⁎⁎⁎	−0.087⁎⁎⁎	−0.086⁎⁎⁎	−0.097⁎⁎⁎	0.043	−0.032	−0.064⁎⁎⁎	−0.071⁎⁎⁎	−0.018	0.005	−0.056⁎⁎⁎	−0.090⁎⁎⁎
HH Land Cultivated	0.001	−0.004	−0.003	−0.002	−0.003	−0.002	0.008⁎⁎	0.007⁎⁎	0.004	0.008	0.004	0.006⁎	0.003
HH Distance Pavement	0.002⁎⁎⁎	0.003⁎⁎⁎	0.002⁎⁎	0.002⁎⁎⁎	0.002⁎	0.002⁎⁎	0.001	0.001⁎	0.003⁎⁎⁎	0.001	0.001	0.002⁎⁎	0.002⁎⁎
HH Children < Age 10	−0.128⁎	−0.251⁎⁎⁎	−0.071	−0.110	−0.085	−0.199⁎⁎	−0.143⁎	−0.082	−0.209⁎⁎⁎	−0.206⁎⁎	−0.241⁎⁎	−0.086	0.156⁎

Age Difference	0.004	0.007	0.001	−0.008	0.001	0.004	0.002	0.003	0.004	0.011⁎	0.009	0.002	0.002
Education Difference	−0.015	0.054	0.051	0.034	0.060	−0.067	0.055	−0.018	−0.013	0.075	0.052	−0.054	0.055
Health Difference	−0.084⁎	−0.034	−0.014	0.017	0.019	−0.135⁎⁎	−0.111⁎⁎	−0.118⁎⁎	−0.022	−0.008	0.019	−0.069⁎	−0.034
Farm Labor Difference	−0.008	−0.063⁎⁎⁎	−0.034	−0.016	−0.016	0.027	−0.007	−0.065⁎⁎⁎	−0.073⁎⁎⁎	−0.054⁎⁎⁎	−0.086⁎⁎⁎	−0.089⁎⁎⁎	−0.002
Market Labor Difference	0.020	0.000	−0.004	−0.025	0.007	0.015	−0.042⁎	0.004	0.013	0.012	−0.003	−0.007	−0.02
Home Labor Difference	0.029	0.049⁎⁎	0.057⁎⁎	0.090⁎⁎⁎	0.073⁎⁎⁎	−0.079⁎⁎⁎	0.055⁎⁎	0.016	0.064⁎⁎⁎	0.072⁎⁎⁎	0.049⁎	0.071⁎⁎⁎	0.102⁎⁎⁎

Eastern	*-omitted-*	*-omitted-*	*-omitted-*	*-omitted-*	*-omitted-*	*-omitted-*	*-omitted-*	*-omitted-*	*-omitted-*	*-omitted-*	*-omitted-*	*-omitted-*	*-omitted-*
Central	−0.297⁎	−0.421⁎⁎	−0.428⁎	−0.902⁎⁎⁎	−0.583⁎⁎	0.584⁎⁎⁎	0.256	−0.083	−0.068	−0.401⁎	−0.100	−0.168	−0.449⁎⁎
Southern Highlands	0.000	0.005	0.070	−0.097	0.165	−0.541⁎⁎⁎	−0.036	0.160	0.318⁎⁎	−0.090	0.299⁎	−0.048	0.293⁎
Lake	0.102	0.059	0.114	−0.271⁎⁎	−0.141	−0.039	0.142	0.307⁎⁎⁎	0.050	−0.062	0.369⁎⁎	0.065	−0.053
Northern	0.046	0.005	0.144	−0.179	0.020	−0.159	0.194	0.122	0.094	0.045	0.328⁎⁎	0.221⁎⁎	0.027
Southern	0.410⁎⁎⁎	0.267⁎⁎	0.217	0.261⁎⁎	0.177	0.312⁎⁎	0.248⁎	0.448⁎⁎⁎	0.434⁎⁎⁎	0.266⁎	0.639⁎⁎⁎	0.408⁎⁎⁎	0.343⁎⁎
Western	0.121	−0.004	−0.212	−0.159	−0.16	0.118	0.432⁎⁎⁎	0.251⁎⁎	0.082	−0.175	0.444⁎⁎⁎	0.11	−0.082

Constant	3.518⁎⁎⁎	3.703⁎⁎⁎	3.257⁎⁎⁎	3.692⁎⁎⁎	3.416⁎⁎⁎	3.739⁎⁎⁎	3.799⁎⁎⁎	4.344⁎⁎⁎	3.291⁎⁎⁎	3.412⁎⁎⁎	3.125⁎⁎⁎	3.870⁎⁎⁎	2.773⁎⁎⁎
R-squared	0.051	0.054	0.047	0.072	0.049	0.061	0.043	0.051	0.068	0.05	0.071	0.072	0.052
N	1750	1719	1436	1720	1429	1746	1681	1715	1736	1568	1448	1755	1745

*Notes*: All regressions control for the sample weight and strata. Differences are always calculated as [Husband response − Wife response]. A positive coefficient on a difference variable implies that as the husband’s response increases relative to the wife’s, or as the wife’s response decreases relative to the husband’s, the number of beans allocated to the wife increases.

⁎ *p* < 0.10.

⁎⁎ *p* < 0.05.

⁎⁎⁎ *p* < 0.01.

**Table 3:** OLS results—number of beans allocated to wives by themselves (W_i_[W_i_]) by decision as a function of wife assets and relative spousal attributes

Models	(1)	(2)	(3)	(4)	(5)	(6)	(7)	(8)	(9)	(10)	(11)	(12)	(13)
	**Farm decisions and cash**					**Children**		**Innovation**				**Livelihood *versus* overall**	
	What crops to grow	Where to sell crops	When to sell livestock	How to use cash from crops	How to use cash from livestock	What foods to feed family	Children’s schooling	Whether to buy new seed	Whether to buy tools	What training needed	Who attends training	Farm generally	Overall authority
Wife Age	0.002	0.015⁎⁎⁎	0.007	0.010⁎⁎⁎	0.008	0.004	0.006	0.001	0.007⁎⁎	0.010⁎⁎	−0.001	0.006⁎	0.015⁎⁎⁎
Wife Education	0.009	0.011	0.011	0.029⁎⁎	0.032⁎	−0.010	0.034⁎⁎	0.000	0.001	0.022	0.021	0.018	0.033⁎⁎
Wife Health	−0.106⁎	0.043	0.015	0.030	−0.029	−0.185⁎⁎	−0.055	−0.111	−0.031	0.116	−0.054	−0.076	0.164⁎⁎
Wife Farm Labor	0.086⁎⁎⁎	0.012	0.056⁎	0.010	0.081⁎⁎	0.049	0.058⁎⁎	0.059⁎⁎	0.060⁎⁎	0.100⁎⁎⁎	0.062⁎	0.029	0.121⁎⁎⁎
Wife Market Labor	0.001	0.054⁎	0.076⁎⁎	0.040	0.007	0.023	−0.038	−0.035	−0.024	−0.022	−0.039	0.014	−0.068⁎⁎
Wife Home Labor	−0.033	−0.028	−0.051	−0.023	−0.015	−0.020	0.014	0.014	0.027	−0.028	−0.055	−0.011	0.030

HH Asset Index	−0.004	−0.002	0.003	0.004	0.004	0.009⁎	−0.007	0.007⁎	−0.002	0.004	0.007	−0.000	0.004
Husband’s Community Standing	−0.005	−0.018	−0.028	−0.046⁎	−0.047	0.020	−0.047⁎	−0.055⁎⁎	0.004	0.005	0.053	−0.056⁎⁎	−0.071⁎⁎⁎
HH Land Cultivated	0.001	0.002	−0.006	0.003	−0.007⁎	−0.001	0.002	−0.004	0.005	−0.004	0.007	−0.001	0.007⁎⁎
HH Distance Pavement	0.001	0.002⁎⁎⁎	0.001	0.001	0.000	0.003⁎⁎⁎	0.002⁎⁎	0.001	0.002⁎⁎	−0.000	−0.002⁎	0.000	0.000
HH Children < Age 10	−0.022	−0.102	−0.082	−0.093	0.023	−0.037	−0.179⁎⁎	−0.091	−0.062	0.035	−0.120	−0.033	0.042

Age Difference	−0.002	0.001	0.000	−0.000	0.012⁎	−0.000	−0.003	0.005	0.003	0.008	−0.006	−0.004	−0.003
Education Difference	0.030	0.005	0.005	0.029	0.054	−0.103⁎	0.064	0.038	−0.002	0.034	0.046	0.017	0.028
Health Difference	−0.128⁎⁎⁎	−0.102⁎	−0.135⁎⁎	−0.066	−0.039	−0.153⁎⁎	−0.040	−0.110⁎⁎	−0.067	0.056	−0.159⁎⁎	−0.030	0.055
Farm Labor Difference	0.041⁎⁎	−0.004	−0.003	0.021	0.078⁎⁎⁎	−0.030	0.034	0.038⁎	0.005	−0.001	0.039	−0.001	0.077⁎⁎⁎
Market Labor Difference	−0.010	−0.003	0.055⁎⁎	−0.011	0.012	0.007	−0.029	−0.022	−0.017	0.001	−0.045⁎	−0.009	−0.040⁎⁎
Home Labor Difference	−0.000	0.007	0.005	0.013	0.033	−0.044	0.013	0.011	0.045⁎⁎	−0.042	−0.044	0.038⁎	0.062⁎⁎⁎

Eastern	*-omitted-*	*-omitted-*	*-omitted-*	*-omitted-*	*-omitted-*	*-omitted-*	*-omitted-*	*-omitted-*	*-omitted-*	*-omitted-*	*-omitted-*	*-omitted-*	*-omitted-*
Central	−0.105	−0.179	−0.319	−0.037	0.215	0.930⁎⁎⁎	0.431⁎	0.532⁎⁎⁎	0.504⁎⁎⁎	−0.195	0.020	0.368⁎	−0.612⁎⁎⁎
Southern Highlands	0.189	0.331⁎⁎	−0.336⁎	−0.241	−0.236	0.374⁎⁎	0.232	0.243	0.456⁎⁎⁎	0.369⁎⁎	0.369⁎⁎	0.403⁎⁎⁎	0.028
Lake	0.193	0.218	0.018	0.179	−0.072	0.069	0.620⁎⁎⁎	0.229⁎	0.364⁎⁎⁎	0.143	0.627⁎⁎⁎	0.311⁎⁎	−0.276⁎⁎
Northern	0.200⁎	0.268⁎⁎	−0.225	0.022	−0.007	0.295⁎	0.700⁎⁎⁎	0.781⁎⁎⁎	0.539⁎⁎⁎	0.075	0.075	0.394⁎⁎⁎	0.086
Southern	0.280⁎⁎⁎	0.305⁎⁎	−0.213	−0.091	−0.136	0.472⁎⁎⁎	0.683⁎⁎⁎	0.533⁎⁎⁎	0.455⁎⁎⁎	0.338⁎⁎	0.213	0.520⁎⁎⁎	−0.345⁎⁎⁎
Western	0.163	0.221⁎	−0.513⁎⁎⁎	−0.235⁎⁎	−0.597⁎⁎⁎	0.312⁎⁎	0.671⁎⁎⁎	0.540⁎⁎⁎	0.373⁎⁎⁎	0.038	0.157	0.461⁎⁎⁎	−0.271⁎⁎

Constant	4.277⁎⁎⁎	2.968⁎⁎⁎	3.732⁎⁎⁎	3.350⁎⁎⁎	3.435⁎⁎⁎	4.644⁎⁎⁎	3.905⁎⁎⁎	4.174⁎⁎⁎	3.241⁎⁎⁎	2.541⁎⁎⁎	3.921⁎⁎⁎	3.867⁎⁎⁎	1.896⁎⁎⁎
R-squared	0.022	0.037	0.033	0.027	0.038	0.041	0.048	0.047	0.033	0.030	0.040	0.029	0.064
N	1754	1705	1483	1709	1481	1741	1687	1710	1722	1526	1420	1752	1729

*Notes*: All regressions control for the sample weight and strata. Differences are always calculated as [Husband response − Wife response]. A positive coefficient on a difference variable implies that as the husband’s response increases relative to the wife’s, or as the wife’s response decreases relative to the husband’s, the number of beans allocated to the wife increases.

⁎ *p* < 0.10.

⁎⁎ *p* < 0.05.

⁎⁎⁎ *p* < 0.01.

**Table 4:** Summary OLS results—significant coefficients for beans allocated to wives by husbands (W_i_[H_i_]) or beans allocated to wives by themselves W_i_[W_i_] by decision

**Table 5:** Intra-household accord (W_*i*_[H_*i*_] − W_*i*_[W_*i*_]) means and variability across decisions

Decision variable	Obs.	Mean accord W*_i_*[H*_i_*] − W*_i_*[W*_i_*]	Std. Dev.	Min	Max	*t*-Test of mean = 0	% Discord (>1 bean)	% Discord (>2 beans)
Crops cultivated	1844	0.009	1.628	−10	8	0.229	63	29
Where to sell crops	1777	0.187	1.867	−9	8	4.219⁎⁎⁎	66	34
When to sell livestock	1444	0.096	1.920	−9	9	1.905⁎	64	34
Cash spending (crops)	1784	0.134	1.825	−8	7	3.100⁎⁎⁎	66	35
Cash spending (livestock)	1443	0.150	2.046	−10	10	2.793⁎⁎⁎	67	37
Food to feed family	1830	−0.128	2.101	−8	7	−2.604⁎⁎⁎	68	39
Schooling	1739	0.001	1.887	−9	9	0.013	60	33
Buy new seeds	1768	−0.000	0.043	−9	7	−0.000	62	31
Buy new equipment	1806	0.131	0.040	−9	7	3.313⁎⁎⁎	64	31
Types info. needed	1528	0.122	2.078	−9	9	2.302⁎⁎	64	38
Who attends training	1385	0.022	2.281	−10	10	0.353	65	41
Farm generally	1848	0.175	1.660	−9	8	4.526⁎⁎⁎	64	30
Overall household	1814	0.302	1.962	−7	9	6.557⁎⁎⁎	68	40

⁎ *p* < 0.10.

⁎⁎ *p* < 0.05.

⁎⁎⁎ *p* < 0.01.

**Table 6:** Logistic regression results for intra-household accord (|W*_i_*[H*_i_*] − W*_i_*[W*_i_*]| < 2) by decision as a function of wife assets and relative spousal attributes (marginal effects)

*Notes*: All regressions control for the sample weight and strata. Differences are always calculated as [Husband response − Wife response]. A positive coefficient on a difference variable implies that as the husband’s response increases relative to the wife’s, or as the wife’s response decreases relative to the husband’s, the likelihood of accord increases.

^*^p < 0.10.

^**^p < 0.05.

^***^p < 0.01.

**Table 7:** Logistic regression for accord (|W_*ijk*_[H_*ijk*_]—W_*ijk*_[W_*ijk*_]| < 2) as a function of household and decision characteristics (marginal effects, clustered by household and decision)

*Notes*: All regressions control for sample weight and strata. Differences are calculated as [Husband response] − [Wife response].

^*^p < 0.10.

^**^p < 0.05.

^***^p < 0.01.

## Footnotes

1 We use the word “authority” as we believe it best reflects a share of power in decision-making that reflects our survey and experimental set-up.

2 Malhotra and Schuler (2005) define women’s empowerment as a process including increased mobility, access to and control over economic resources and domestic decision-making.

3 Because household decisions are often repeated, one criticism of non-co-operative models is that a co-operative outcome should evolve; that is, spouses should “figure out” how to arrive at optimal solutions, especially for providing household public goods. Evidence, however, suggests the contrary, with strategic and non-co-operative behavior commonly observed even in decisions surrounding shared household goods (such as children’s nutrition and education).

4 We chose to look at the share of authority given to wives, rather than the share given to husbands, on the assumption that the husband is the default head of the household and cedes authority to his wife, rather than the other way around. Indeed, in our sub-sample of married couples only rarely is the woman designated as the “head of household” or primary decision-maker.

5 Data for the study were collected as a part of the 2010 Farmer Focus survey conducted by the Agricultural Development Program of the Bill & Melinda Gates Foundation.

6 Note that since authority is measured by the share of ten beans that each spouse accords themselves, this measure of accord is symmetric with (mirrors) the husband’s self-reported authority and his authority as reported by the wife.

7 Another possible interpretation of these findings is that as the husband’s health worsens relative to the wife—as in the case of HIV/AIDS infection—the wife’s claim to authority increases. Although our data do not allow direct testing of this hypothesis, some of the observed regional effects may reflect variation in the regional prevalence of HIV/AIDs (Tanzania Demographic and Health Survey (TDHS), 2010).
